# Activation of a GPCR, ORL1 Receptor: A Novel Therapy to Prevent Heart Failure Progression

**DOI:** 10.3390/jcdd11110355

**Published:** 2024-11-05

**Authors:** Saliha S. Pathan, Aarthi Pugazenthi, Beverly R. E. A. Dixon, Theodore G. Wensel, Todd K. Rosengart, Megumi Mathison

**Affiliations:** 1 Michael E. DeBakey Department of Surgery, Baylor College of Medicine, One Baylor Plaza, Houston, TX 77030, USA; saliha.pathan@bcm.edu (S.S.P.); aarthikp@gmail.com (A.P.); brdixon1@mdanderson.org (B.R.E.A.D.); todd.rosengart@bcm.edu (T.K.R.); 2 Department of Biochemistry and Molecular Biology, Baylor College of Medicine, One Baylor Plaza, Houston, TX 77030, USA; twensel@bcm.edu

**Keywords:** prevention of ischemic heart failure progression, ORL1 receptor activation, MCOPPB

## Abstract

The number of ischemic heart failure (HF) patients is growing dramatically worldwide. However, there are at present no preventive treatments for HF. Our previous study showed that Gata4 overexpression improved cardiac function after myocardial infarction in rat hearts. We also found that Gata4 overexpression significantly increased the expression of a Pnoc gene, an endogenous ligand for the cell membrane receptor ORL1. We hypothesized that the activation of the ORL1 receptor would suppress HF in a rat ischemic heart model. Adult Sprague Dawley rats (8 weeks old, six males and six females) underwent left anterior descending coronary artery ligation. Three weeks later, normal saline or MCOPPB (ORL1 activator, 2.5 mg/kg/day) intraperitoneal injection was started, and continued 5 days a week for 3 months. Echocardiography was performed six times: pre-operative, 3 days after coronary artery ligation, pre-MCOPPB or saline injection, and 1, 2, and 3 months after saline or MCOPPB injection started. Animals were euthanized after 3 months’ follow-up and the hearts were harvested for histological analysis. The ORL1 activator, MCOPPB, significantly improved cardiac function after myocardial infarction in rats (ejection fraction, MCOPPB vs. saline at euthanasia, 67 ± 3% vs. 43 ± 2%, *p* < 0.001). MCOPPB also decreased fibrosis and induced angiogenesis. Thus, the ORL1 activator, MCOPPB, may be a novel treatment for preventing HF progression.

## 1. Introduction

During the past two decades, we and several other researchers have reported that Gata4 overexpression (OE) by direct injection into the heart muscle improved cardiac function after myocardial infarction (MI) in the murine heart [1,2,3]. Dr. Ruskoaho’s group [4] also reported that Gata4 reduced angiotensin-induced remodeling in rat hearts by interfering with pro-fibrotic and hypertrophic gene expression. Despite those positive reports, Gata4 OE has not yet been pursued as a heart failure (HF) treatment. Since Gata4 is an essential transcription factor in the gene regulation of cardiac hypertrophy, recent research has been focused on targeting Gata4 as a modulator of post-translational modification [5].

**Figure 1:**
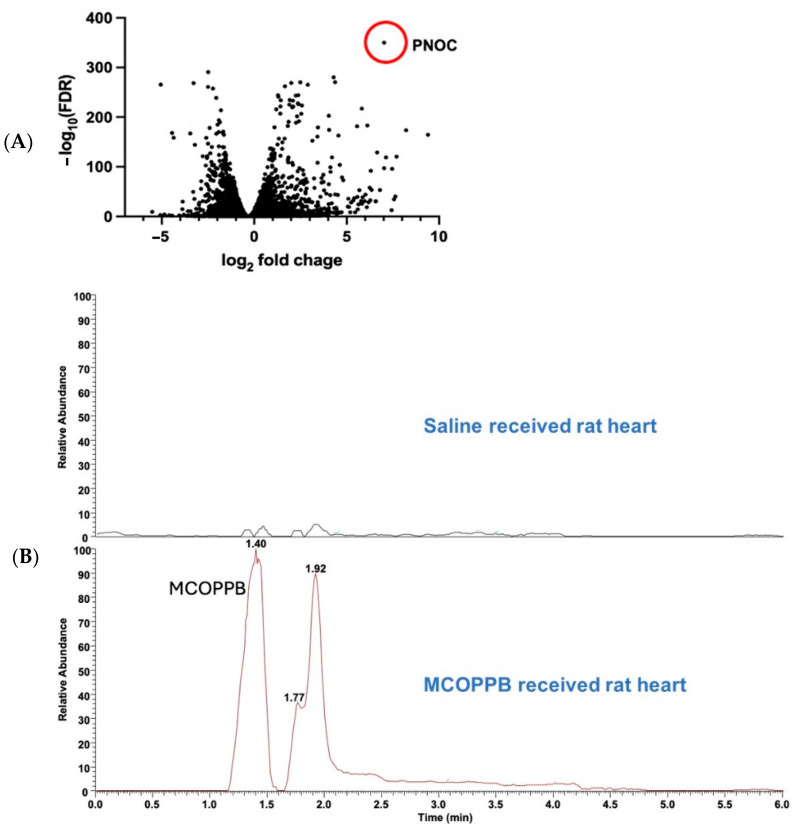

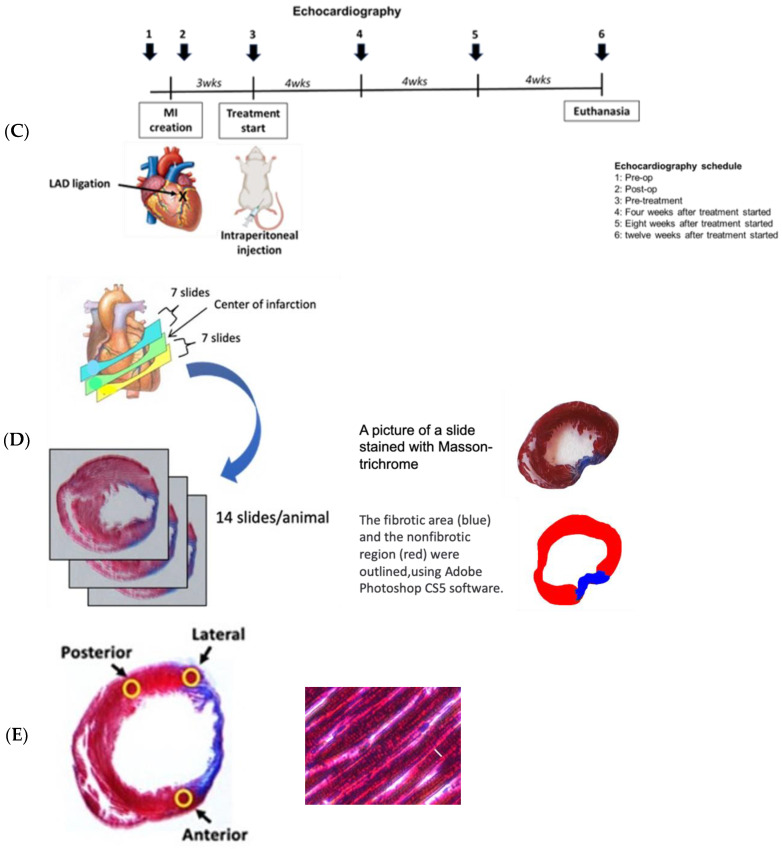
(**A**) Volcano plot of RNAseq analysis. Cardiac fibroblasts were treated with lentivirus encoding Gata4 or GFP (each 20 MOI) for 14 days (*n* = 3). PNOC gene was significantly upregulated by Gata4. (**B**) Chromatography assay for MCOPPB detection in the heart. Rats received MCOPPB 2.5 mg/kg/day or saline intraperitoneally for 4 days (*n* = 3/group) and were euthanized immediately after the last injection. The heart tissues were examined with mass spectrometry. MCOPPB was detected in the heart tissue of all MCOPPB-receiving rats but no MCOPPB in the saline-receiving rats. Representative chromatography of an MCOPPB-receiving rat is shown. (**C**) Schematic showing the experimental design of the MCOPPB study. Twelve adult Sprague Dawley rats were enrolled. Three weeks after the coronary artery ligation, they were treated with saline or MCOPPB for 3 months; 6 rats (3 males and 3 females) for saline, and another 6 rats (3 males and 3 females) for MCOPPB. Echocardiography was performed six times, 1: pre-op, 2: post-op, 3: pre-treatment, 4: four weeks after treatment started, 5: eight weeks after treatment started, and 6: twelve weeks after treatment started. (**D**) Fibrosis analysis. The excised heart was cut transversally and sectioned with two (2 to 3 mm thick) slices obtained, with one immediately cephalad and another one immediately caudad to the transverse centerline of the infarct region, which was readily identifiable by gross inspection. After paraffin embedding of these slices, 14 sections per animal (at a 120 μm interval between each section) were stained with Masson’s trichrome to assess the extent of fibrosis (**D**). The fibrotic area (blue) and the nonfibrotic region (red) were outlined in the LV wall including the septum, using Adobe Photoshop CS5 software, version 22.1.1. (**D**) and then quantified with MATLAB and Simulink software, version 7.12.0.635 (MathWorks, Inc., Natick, MA, USA). The percent fibrosis was calculated as: (total of blue pixels from all sections/total of blue plus red pixels from all sections) × 100. (**E**) Three regions where cardiomyocyte diameter was measured: The slide demonstrating the greatest area of fibrosis, as identified by Masson’s Trichrome staining, was selected for each animal. In each slide, 20 longitudinally oriented cardiomyocytes from each of the 3 regions, anterior, lateral, and posterior, were examined, and the diameters were defined. The mean value of 20 measurements represented 1 sample from each position in each animal. A representative photo shows how to measure with a white bar.

After we reported that Gata4 OE suppressed HF [3], we further investigated the effects of Gata4 OE in vitro. Our RNAseq analysis revealed that the Pnoc gene was significantly upregulated by Gata4 OE in cardiac fibroblasts (Figure 1A and Table 1). Pnoc is a preproprotein and is proteolytically processed to generate nociceptin, which binds to ORL1. ORL1 belongs to the G protein-coupled receptor (GPCR) and is the most recently discovered member of the opioid receptor family [6]. Nociceptin and ORL1 have been intensely investigated in the neuroscience field [6], and their cardiovascular effects have also been characterized. Similar to opioids, nociceptin causes hypotension without reflex tachycardia after acute intravenous injection [7]. Gumusel B et al. reported that nociceptin has vasorelaxant properties on peripheral artery rings, suggesting that nociceptin possesses biological activity outside the central nervous system and may contribute to the regulation of systemic blood pressure and regional blood flow [8]. Simonsen U et al. showed that ZP120, partial ORL1 agonist, and nociceptin have a vasodilatory effect by the prejunctional inhibition of adrenergic neurotransmission [9]. The authors state that ZP120 can attenuate HF by decreasing sympathetic activity which may lead to the deterioration of cardiac pump function by eliciting peripheral vasoconstriction. However, a clinical trial of ZP120 for congestive HF patients was terminated due to significant hypotensive activity [10]. Then, Dr. Hobbs’ group investigated SER100 (previously known as ZP120) in vivo, using spontaneously hypertensive rats and hypoxia-induced pulmonary hypertension models. They showed that SER100 exerts chronic hypotensive and bradycardic effects in rodents, including models of systemic and pulmonary hypertension, and concluded that the cardiovascular effects produced by SER100 were, at least in part, due to the inhibition of cardiac and vascular sympathetic activity, and the blood pressure-lowering effects of SER100 can be applied as a treatment for hypertension [10].

In this study, we investigated whether the activation of ORL1 without Gata4 administration improved cardiac function after myocardial infarction, thus inhibiting HF progression. We demonstrated that the administration of the ORL1 activator, MCOPPB, improved cardiac function after myocardial infarction in rats. Although there are many questions to be answered, such as whether the cardiac improvement is through the nervous systems or as a result of molecular biological changes directly inside the cell, the results in this study are encouraging and warrant further investigation.

## 2. Materials and Methods

### 2.1. Animals

All animal experiments were performed in accordance with the IACUC protocol and approved by the Baylor College of Medicine (no. AN-6223). Animals were housed and cared for in facilities run by the Center for Comparative Medicine at Baylor College of Medicine, which is fully accredited by the Association for the Assessment and Accreditation of Laboratory Animal Care International. Adult Sprague Dawley rats (Inotiv Co., West Lafayette, IN, USA) (8 weeks old) underwent left anterior descending (LAD) coronary artery ligation (described in detail in our previous publication [11]). In brief, animals were first anesthetized with Isoflurane 4% in an induction box, intubated, and placed on a rodent ventilator (Harvard Apparatus, Holliston, MA, USA) using Isoflurane inhalation (3%) supplemented with oxygen. Meloxicam (2 mg/kg) and Buprenorphine Extended Release (1 mg/kg) were administered subcutaneously. Bupivacaine, as a 50/50 mix with Lidocaine, with 0.1 mL of 1:20 dilution, was given by subcutaneous application along the incision line. A left thoracotomy was then performed, and the left coronary artery was ligated 1 to 2 mm from its origin with a 7-0 polypropylene suture. All animals received post-operative pain medication, Meloxicam (2 mg/kg) for 5 days, and Buprenorphine Extended Release (1 mg/kg) at post-operative day 3. Two males and three females died due to myocardial infarction. The final enrolled animal numbers were 6 males and 6 females.

### 2.2. ORL1 Activator

We selected MCOPPB (Sigma-Aldrich, Saint Louis, MO, USA, PZ0159) as an ORL1 receptor activator. MCOPPB is reported to be the most potent and novel non-peptide ORL1 full agonist drug in vitro [12]. A dosage of MCOPPB of 2.5 mg/kg/day was chosen based on the article published by Raffaele M et al. [13]. They administered MCOPPB at a rate of 5 mg/kg /day to mice by intraperitoneal injection. According to the guide for dose conversion between animals [14], we administered MCOPPB at a rate of 2.5 mg/kg/day to the rats. MCOPPB was dissolved in normal saline. In order to confirm that MCOPPB was delivered to the heart with this dosage, we tested the administration of 2.5 mg/kg/day MCOPPB or normal saline intraperitoneally in 3 rats/group for 4 days after coronary artery ligation and the heart was harvested. Mass spectrometry confirmed MCOPPB upregulation in the heart of MCOPPB-receiving rats, but no MCOPPB upregulation was observed in the heart of saline-receiving rats (Figure 1B).

### 2.3. Treatment

Three weeks after the coronary artery ligation, normal saline or MCOPPB (2.5 mg/kg/day) intraperitoneal injection was started, and continued 5 days per week for 3 months (Figure 1C). Body weight was measured every Monday after the administration started and at euthanasia.

### 2.4. Echocardiography

Echocardiography (Vevo 770 Imaging System, VisualSonics, Toronto, ON, Canada) was performed six times: pre-operative, 3 days after coronary artery ligation, pre-MCOPPB or saline injection, and 1, 2, and 3 months after saline or MCOPPB injection started (Figure 1C). Animals were sedated during the echocardiography with Isoflurane 2.5%. Echo images of the left ventricle were obtained in short-axis views by investigators blinded to the treatment groups. Left ventricular end-systolic and end-diastolic diameters and left ventricular septal and posterior thickness (in both the end-systolic and end-diastolic phases) were measured from M-mode tracings. These imaging data were then analyzed by investigators blinded to the treatment groups. 

### 2.5. Euthanasia and Histological Analysis

After 3 months’ follow-up, animals were euthanized with bilateral opening of the chest and exsanguination under surgical plane of anesthesia with Isoflurane 4% and the heart was harvested. The body weight and the heart weight were measured. For the histological analysis, the excised heart was fixed with 4% paraformaldehyde for 24 h and then 2% paraformaldehyde for 48 h at 4 °C. The heart was then cut transversally and sectioned with 2 (2 to 3 mm) slices obtained, one immediately cephalad and the other one immediately caudad to the transverse centerline of the infarct region, which was readily identifiable by gross inspection (Figure 1D). After paraffin embedding of these slices, 14 sections per animal (at a 120 μm interval between each section) were stained with Masson’s Trichrome to assess the extent of fibrosis (Figure 1D). The fibrotic area (blue) and the nonfibrotic region (red) were outlined in the LV wall, including the septum, using Adobe Photoshop CS5 software (Figure 1D), and then quantified with MATLAB and Simulink software (MathWorks, Inc., Natick, MA, USA). The percentage of fibrosis was calculated as: (total of blue pixels from all sections/total of blue plus red pixels from all sections) × 100.

Cardiomyocyte diameter was measured at 400× magnification of cardiomyocytes found in the peri-infarct (anterior, lateral) regions subtended by the ligated left anterior descending coronary artery and the non-infarcted (posterior) LV regions (Figure 1E). The slide demonstrating the greatest area of fibrosis, as identified by Masson’s Trichrome staining, was selected for each animal. In each slide, 20 longitudinally oriented (long-axis) cardiomyocytes from each of the 3 regions, anterior, lateral, and posterior, were examined, and the diameters were defined. The mean value of 20 measurements represented 1 sample from each position in each animal.

For angiogenesis analysis, two sections per animal, in which infarction size was largest in the transverse section, were stained with CD31 (R&D systems, Minneapolis, MN, USA, AF3628). First, the stained sections were searched for CD31-positive cells by five random microscopic fields per slide at ×200 magnification and the highest number identified in the peri-infarct region was chosen as the number of CD31-positive cells for each slide. EVOS (Brisbane, Australia) M5000 microscopy was used for immunohistochemical analysis.

### 2.6. Randomization and Blinding

Rats were randomly allocated to the experimental groups, with 3 males and 3 females in each group: saline and MCOPPB. All experiments, including animal surgery, echocardiography, and echocardiographic and histological analyses were conducted by researchers blinded to the treatment groups.

### 2.7. Preliminary In Vitro Experiment

We performed preliminary in vitro experiments to find the molecular biological aspects of heart failure suppression by ORL1 activation.

(1) Primary adult rat brain tissue lysates, adult rat cardiac fibroblasts, neonatal rat cardiomyocytes (p2), and H9C2 cells (ATCC-CRL-1446) were immunostained with anti-nociceptin receptor antibody (alomone labs, Jerusalem, Israel, AOR-015). Another set of the four cell groups were pre-incubated with ORL1-blocking peptide (alomone labs, BLP-OR015).
(2) Neonatal rat cardiomyocytes (p4) were isolated and treated with 0.5 μM of MCOPPB, followed by the addition of ET-1 after 24 h at 100 nM and the addition of Anti-ORL1 (ORL1 antagonist, [Nphe1]Nociceptin(1-13)NH2], R&D systems, Minneapolis, MN, USA) (10 μM) after 30 min. At day 3, cells were lysed, and RNA was extracted using Qiagen’s RNeasy Mini Kit (74106). CDNA was made using iScript™ Reverse Transcription Supermix for RT-qPCR from Bio-Rad (South Granville, Sydney, 1708840). qPCR was set up using SYBR dye from Bio-rad (1725121) and Primers from Sigma Aldrich. Primers for NPPA: forward, 5′-CGTATACAGTGCGGTGTCCAAC-3′. reverse, 5′-CATCTTCTCCTCCAGGTGGTCTAG-3′. Primers for NPPB: forward, 5′-AAGTCCTAGCCAGTCTCCAGAACA-3′. Reverse, 5′-TTGAGAGCTGTCTCTGAGCCATT-3′. Fold change was calculated after normalization with Gapdh.
(3) H9C2 cells (ATCC-CRL-1446) were treated with 0.5 μM of MCOPPB, followed by the addition of ET-1 after 24 h at 100 nM and the addition of Anti-ORL1 after 30 min at 10 μM. At day 3, cells were harvested by trypsinization using 0.25% trypsin EDTA (Gibco™, Grand Island, NY, USA, 25200056). Cytoplasmic and nuclear proteins were extracted using an extraction kit from BsoterBio (Pleasanton, CA, USA, AR0106). The samples were then quantified for their protein content using a Pierce BCA protein assay kit (Thermo Fisher Scientific, Waltham, MA, USA, 23227). Equal volumes of protein—100 μg—were loaded in each well of a 4–20% gradient gel for SDS-PAGE. The bands were transferred to a nitrocellulose membrane (Invitrogen^TM^, Carlsbad, CA, USA, IB33001) with the help of Iblot3. After blocking the membrane in Bio-Rad Blocking grade buffer (1706404), the membranes were incubated with the primary antibody at a dilution of 1:100 overnight NFATc4 (Santa Cruz Biotechnology, Dallas, TX, USA, SC 271597), Lamin B (loading control) (Santa Cruz Biotechnology—SC 374015). Signals were detected using a horseradish peroxidase-conjugated secondary antibody (CST 7076 or CST 7074) and IMMOBILON forte from Millipore sigma, Darmstadt, Germany (WBLUF0500).
(4) Neonatal mouse cardiomyocytes (p3) were isolated and treated with 0.5μM of MCOPPB at day 1 and day 2. The cells were harvested at day 3 and incubated with the primary antibody, Troponin T (Thermo Fisher Scientific, Rockford, IL, USA, MA5-12960) at a dilution of 1:300 overnight. Cardiomyocyte imaging was conducted for the whole well (15,000 cells per well in a black-walled, glass-bottom 96-well plate) using the high-content imaging instrument Cytation 7 (BioTek, Winooski, VT, USA).

### 2.8. Statistical Analysis

Statistical analysis was performed with SAS version 9.4. The data were presented as mean ± SD. ANOVA was performed to detect statistical significances between multiple groups. When the ANOVA showed significance, a two-tailed ANOVA with a Bonferroni post hoc test was performed. Values of *p* < 0.05 were considered statistically significant.

## 3. Results 

### 3.1. In Vivo Experiments

We did not observe any significant behavioral and physical changes during the follow-up period.

(1) Echocardiography data (raw data can be found in Supplemental Excel file, Table S1).

**Figure 2:**
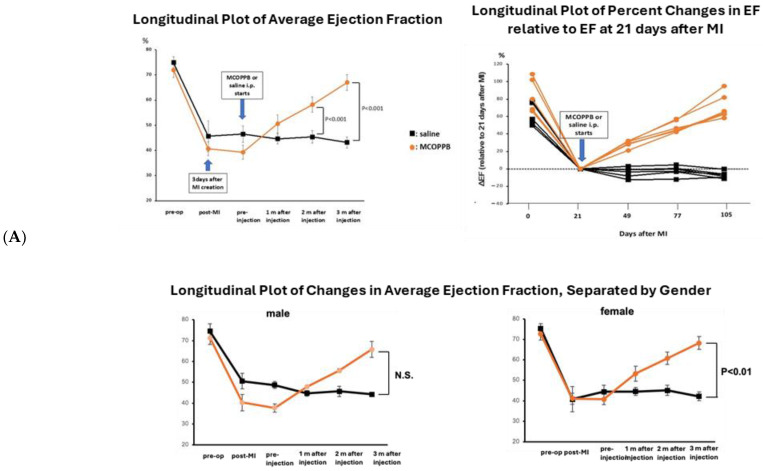

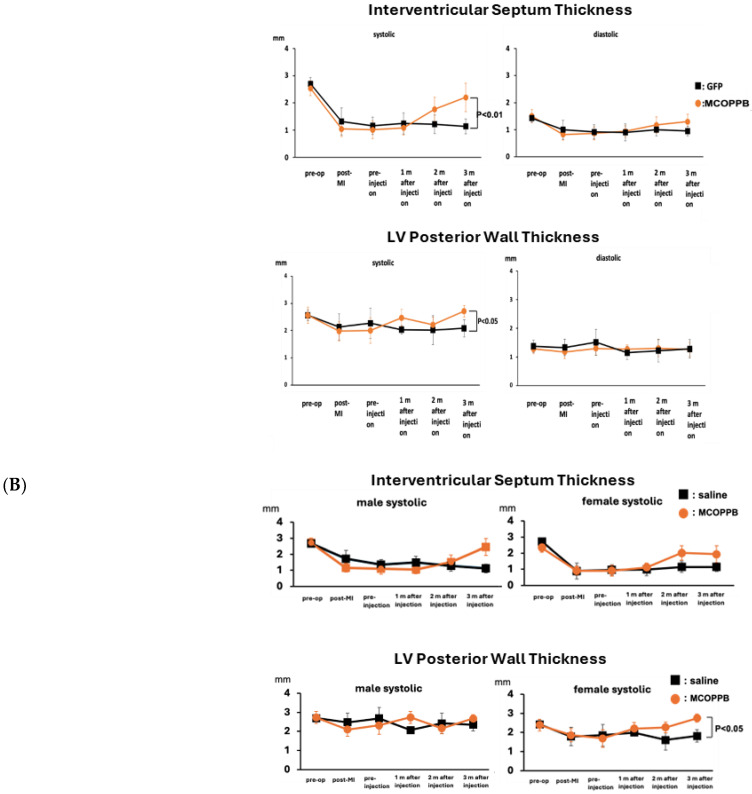

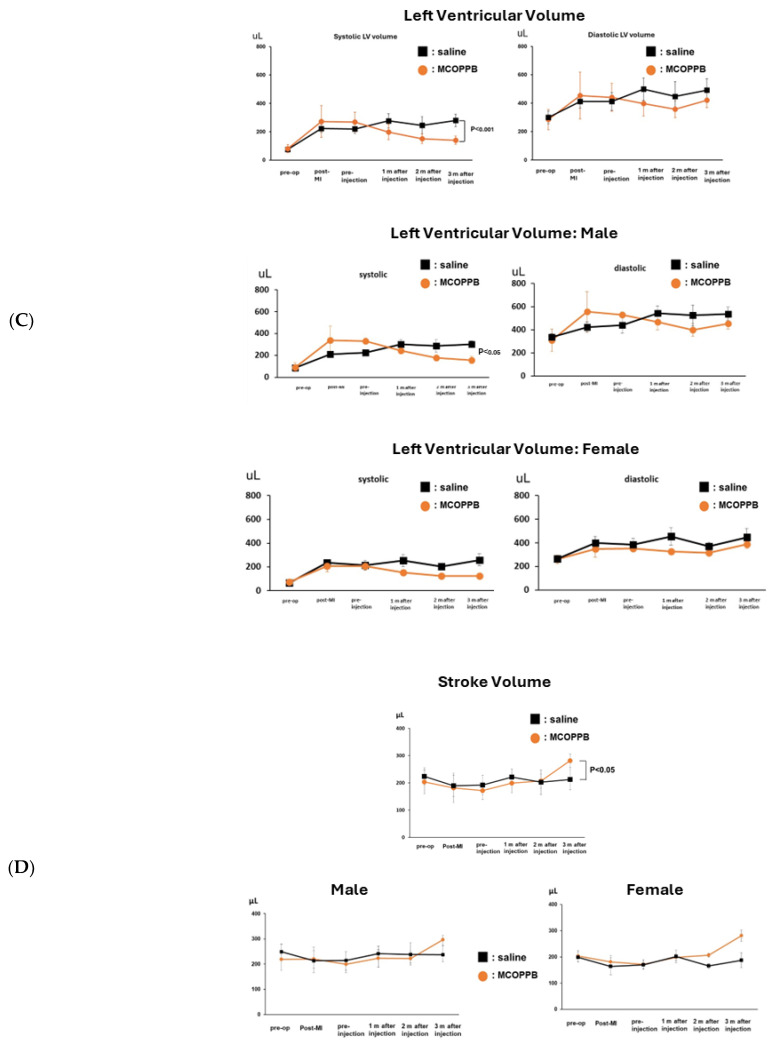

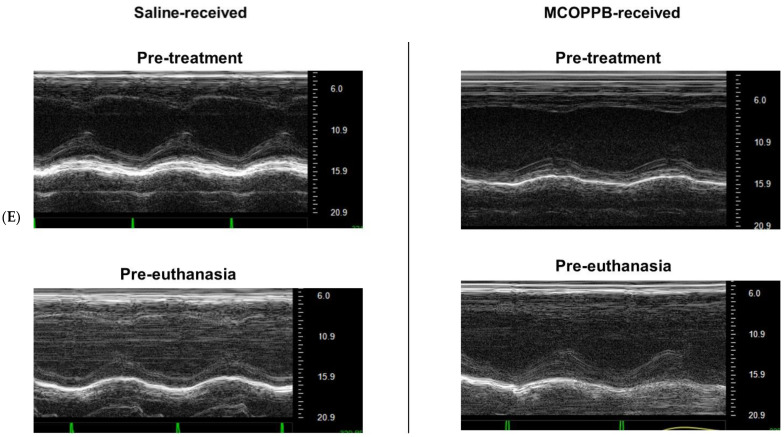
(**A**) Cardiac function was improved with MCOPPB administration. Echocardiography results show longitudinal plot of changes in average ejection fraction (graph on left) and individual relative percent changes in ejection fraction (graph on right) associated with saline (black line) and MCOPPB (orange line) administration, started at day 21 after myocardial infarction induction. Ejection fraction significantly improved in the MCOPPB group (MCOPPB vs. saline at 2 months follow-up, 58 ± 3 vs. 45 ± 2, *p* < 0.001, MCOPPB vs. saline at euthanasia, 67 ± 3 vs. 43 ± 2, *p* < 0.001). Two-tailed ANOVA with Bonferroni post hoc test was used. *n* = 6/group. (**B**) Systolic wall thickness increased in the MCOPPB group. LV wall thickness was measured by echocardiography with short-axis M-mode images at the interventricular wall (left) and posterior wall (right). Both the end-systolic interventricular septum (IVS) (left) and end-systolic left ventricular posterior wall (LVPW) (right) were significantly greater in the MCOPPB group compared to the saline group at euthanasia (end-systolic IVS, MCOPPB vs. saline, 2.2 ± 0.5 mm vs. 1.1 ± 0.3 mm, *p* < 0.01; end-systolic LVPW, MCOPPB vs. saline, 2.7 ± 0.2 mm vs. 2.1 ± 0.3 mm, *p* < 0.05). A two-tailed ANOVA with Bonferroni post hoc test was applied. *n* = 6/group. Longitudinal changes in average systolic interventricular septum thickness and average systolic LV posterior wall thickness were further plotted, separated by gender. LV posterior wall thickness was higher in the female MCOPPB group at euthanasia (MCOPPB vs. saline at euthanasia, 2.7 ± 0.3 vs. 1.8 ± 0.2, *p* < 0.05). A two-tailed ANOVA with Bonferroni post hoc test was used. *n* = 3/group. Orange line: MCOPPB group; black line: saline group. (**C**) End-systolic volume was significantly decreased in the MCOPPB-receiving group at euthanasia. End-systolic volume was calculated with VenoLAB software (comes pre-packaged for applications in the echomachine, Vevo 770 Imaging System): (7.0/(2.4 + LVIDs)) × LVIDs^3^, LVIDs = left ventricular internal diameter end systole). End-systolic volume was significantly decreased in the MCOPPB-receiving group at euthanasia (MCOPPB vs. saline, 140 ± 30 μL vs. 280 ± 44 μL, *p* < 0.001). A two-tailed ANOVA with Bonferroni post hoc test was used. *n* = 6/group. Longitudinal changes in average left ventricular systolic volume and average diastolic volume were further plotted, separated by gender. Systolic left ventricular volume was lower in the male MCOPPB group at euthanasia (MCOPPB vs. saline at euthanasia, 157 ± 32 vs. 300 ± 31, *p* < 0.05). A two-tailed ANOVA with Bonferroni post hoc test was used. *n* = 3/group. Orange line: MCOPPB group; black line: saline group. (**D**) Stroke volume was significantly increased in the MCOPPB-receiving group at euthanasia. Stroke volume was calculated as [(end-diastolic volume) − (end-systolic volume)]. Stroke volume was significantly increased in the MCOPPB-receiving group at euthanasia (MCOPPB vs. saline, 281 ± 25 μL vs. 213 ± 37 μL, *p* < 0.05). Two-tailed ANOVA with Bonferroni post hoc test was used. *n* = 6/group. Longitudinal changes in average left ventricular systolic volume and average diastolic volume were further plotted. (**E**) Representative images obtained by M-mode echocardiography. Left: saline received animal at pre-treatment and pre-euthanasia. Right: MCOPPB received animal at pre-treatment and pre-euthanasia.

Echocardiographic data showed that the ejection fraction significantly improved in the MCOPPB group (MCOPPB vs. saline at 2 months’ follow-up, 58 ± 3 vs. 45 ± 2, *p* < 0.001; MCOPPB vs. saline at euthanasia, 67 ± 3 vs. 43 ± 2, *p* < 0.001) (Figure 2A). Further, measurements of wall thickness by echocardiography showed that both the end-systolic left ventricular posterior wall (LVPW) and end-systolic interventricular septum (IVS) were significantly greater in the MCOPPB group compared to the saline group at euthanasia (end-systolic LVPW, MCOPPB vs. saline, 2.7 ± 0.2 mm vs. 2.1 ± 0.3 mm, *p* < 0.05; end-systolic IVS, MCOPPB vs. saline, 2.2 ± 0.5 mm vs. 1.1 ± 0.3 mm, *p* < 0.05) (Figure 2B). End-diastolic wall thickness showed no difference between the MCOPPB and saline groups (Figure 2B). End-systolic volume was significantly decreased in the MCOPPB-receiving group at euthanasia (MCOPPB vs. saline, 140 ± 30 μL vs. 280 ± 44 μL, *p* < 0.001) while end-diastolic volume showed no difference between the MCOPPB group and saline group (Figure 2C). Stroke volume increased in the MCOPPB group compared to the saline group at euthanasia (MCOPPB vs. saline, 281 ± 250 μL vs. 213 ± 37 μL, *p* < 0.05) (Figure 2D).

(2) Body weight and heart weight analysis.

**Figure 3:**
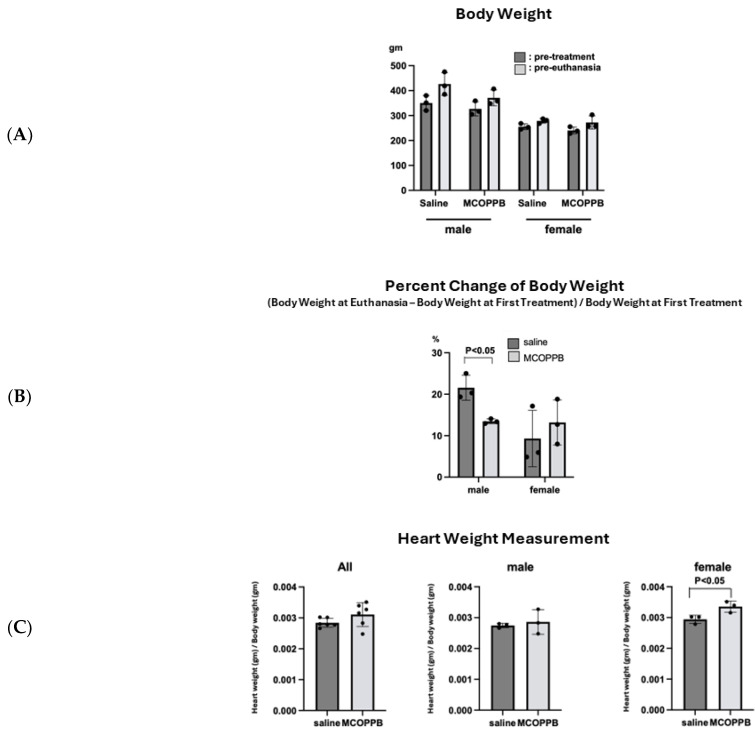
(**A**) Body weight was increased in both the saline- and MCOPPB-receiving groups. Body weight was compared between pre-treatment and pre-euthanasia. All four groups, (1) males receiving saline, (2) males receiving MCOPPB, (3) females receiving saline, and (4) females receiving MCOPPB, gained weight. No changes were statistically significant. (**B**) The percentage of body weight gain was lower in the MCOPPB group than the saline group for males. Body weight change was compared via percentage increase in body weight, [(body weight at euthanasia − body weight at first treatment)/body weight at first treatment] × 100; the male MCOPPB group gained weight less than the male saline group (male saline group, 22 ± 3; male MCOPPB group, 13 ± 0.6; female saline group, 9 ± 7; female MCOPPB group, 13 ± 5) (male saline vs. male MCOPPB, *p* < 0.05). (**C**) Adjusted heart weight by body weight was increased in MCOPPB-receiving animals. The whole heart was analyzed. The heart was harvested after euthanasia and weight was measured. Then, the weight was adjusted by body weight (heart weight/body weight). Adjusted heart weight was increased in MCOPPB-receiving animals in all groups and the increase in the female MCOPPB group was statistically significant (saline group, 0.0029 ± 0.0001; MCOPPB group, 0.0034 ± 0.0002, *p* < 0.05) (**C**).

A body weight comparison between pre-treatment and pre-euthanasia showed that all four groups, (1) males receiving saline, (2) males receiving MCOPPB, (3) females receiving saline, and (4) females receiving MCOPPB, gained weight (Figure 3A). When comparing the percent increase in body weight, [(body weight at euthanasia − body weight at first treatment)/body weight at first treatment] × 100, the male MCOPPB group gained weight less than the male saline group (male saline group, 22 ± 3; male MCOPPB group, 13 ± 0.6; female saline group, 9 ± 7; female MCOPPB group, 13 ± 5) (male saline vs. male MCOPPB, *p* < 0.05) (Figure 3B).

Heart weight adjusted by body weight was increased in the MCOPPB group (saline group, 0.0028 ± 0.0001; MCOPPB group, 0.0031 ± 0.0004) (Figure 3C). Although it was not statistically significant (*p* = 0.15), the adjusted heart weights of females were significantly increased in the MCOPPB group (*p* < 0.05).

(3) Histological analysis.

**Figure 4:**
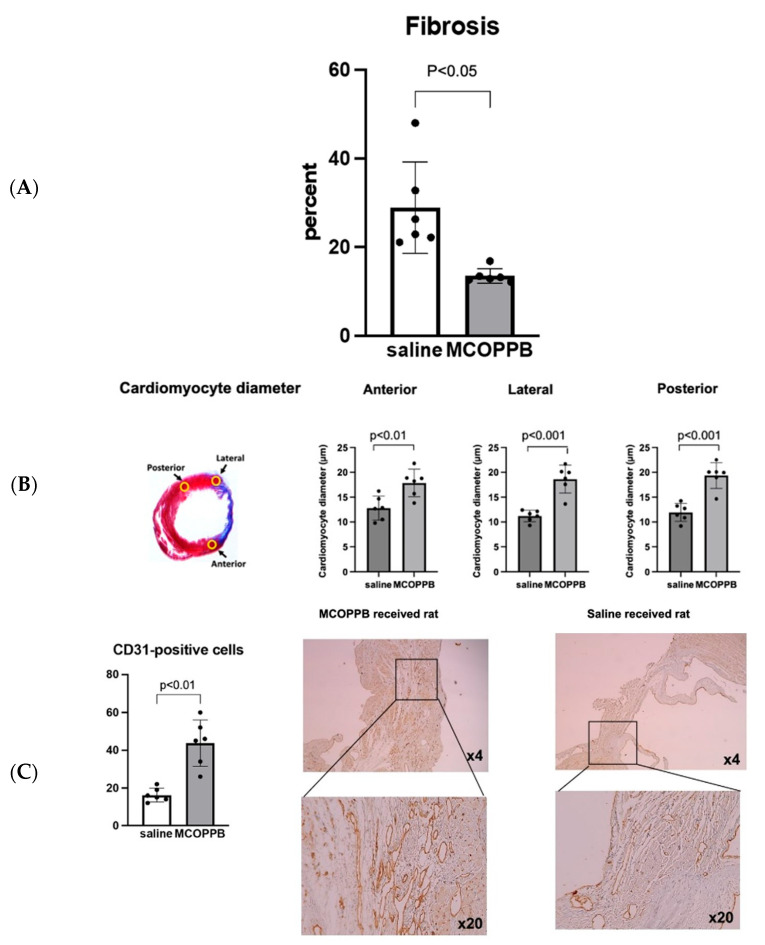
(**A**) MCOPPB decreased the extent of left ventricular wall fibrosis. The paraformaldehyde-fixed heart was cut transversally and sectioned with 2 (2 to 3 mm) slices obtained: one immediately cephalad and the other one immediately caudad to the transverse centerline of the infarct region, which was readily identifiable by gross inspection (Figure 1D). After paraffin embedding of these slices, 14 sections per animal (at a 120 μm interval between each section) were stained with Masson’s Trichrome to assess the extent of fibrosis. Representative images are shown in Figure 1D. The fibrotic area (blue) and the nonfibrotic region (red) were outlined in LV wall including the septum, using Adobe Photoshop CS5 software, (Figure 1D) and then quantified with MATLAB and Simulink software (MathWorks, Inc.). The percentage of fibrosis was calculated as: (total of blue pixels from all sections/total of blue plus red pixels from all sections) × 100. The fibrosis area significantly decreased in the MCOPPB group (% fibrosis area, MCOPPB vs. saline, 14 ± 2 vs. 29 ± 10, *p* < 0.05). A two-tailed *t*-test was used. (**B**) Cardiomyocyte diameter increased in the MCOPPB group. Cardiomyocyte diameter was measured at 400x magnification of cardiomyocytes found in the peri-infarct (anterior, lateral) regions subtended by the ligated left anterior descending coronary artery and the non-infarcted (posterior) LV regions (Figure 1E). The slide demonstrating the greatest area of fibrosis, as identified by Masson’s Trichrome staining, was selected for each animal. In each slide, 20 longitudinally oriented (long-axis) cardiomyocytes from each of the 3 regions, anterior, lateral, and posterior, were examined, and the diameters were defined. The mean value of 20 measurements represented 1 sample from each position in each animal. The left image indicates 3 counting positions (20 cardiomyocytes/position). (MCOPPB vs. saline; anterior, 18 ± 3 μm vs. 13 ± 2 μm, *p* < 0.01, lateral, 19 ± 3 μm vs. 11 ± 1 μm, *p* < 0.001, posterior, 19 ± 3 μm vs. 12 ± 2 μm, *p* < 0.001). The right graphs depict cardiomyocyte diameter quantification. (**C**) MCOPPB increased angiogenesis in infarcted hearts. For angiogenesis analysis, two sections per animal, in which infarction size was largest in the transverse section, were stained with CD31 (R&D systems, AF3628). First, the stained sections were searched for CD31-positive cells by five random microscopic fields per slide at ×200 magnification and the highest number identified in the peri-infarct region was chosen as the number of CD31-positive cells for each slide. EVOS M5000 microscopy was used for immunohistochemical analysis. The MCOPPB group had significantly higher vessel counts in the border zone (44 ± 12 vs. 16 ± 4, *p* < 0.01). The graph shows the number of vessels/field. *n* = 6/group. A two-tailed *t*-test was used. Photo-images are representative slides from the saline and MCOPPB groups.

The fibrosis area, stained by Masson’s Trichrome, significantly decreased in the MCOPPB group (% fibrosis area, MCOPPB vs. saline, 14 ± 2 vs. 29 ± 10, *p* < 0.05) (Figure 4A). Cardiomyocyte diameter was significantly increased in the MCOPPB-receiving group (MCOPPB vs. saline; anterior, 18 ± 3 μm vs. 13 ± 2 μm, *p* < 0.01, lateral, 19 ± 3 μm vs. 11 ± 1 μm, *p* < 0.001, posterior, 19 ± 3 μm vs. 12 ± 2 μm, *p* < 0.001) (Figure 4B). Angiogenesis was assessed by CD31 staining and the MCOPPB group had significantly higher vessel counts in the border zone (44 ± 12 vs. 16 ± 4, *p* < 0.01) (Figure 4C).

### 3.2. In Vitro Experiments

**Figure 5:**
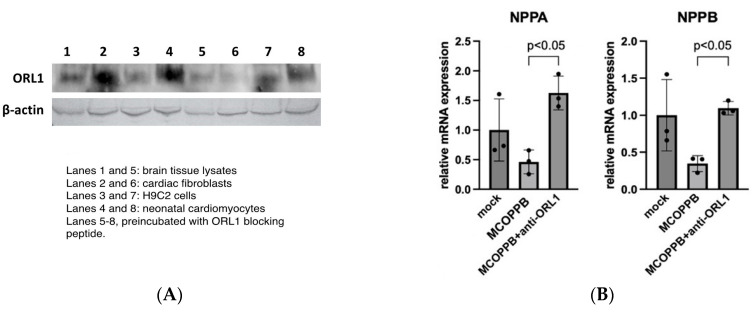

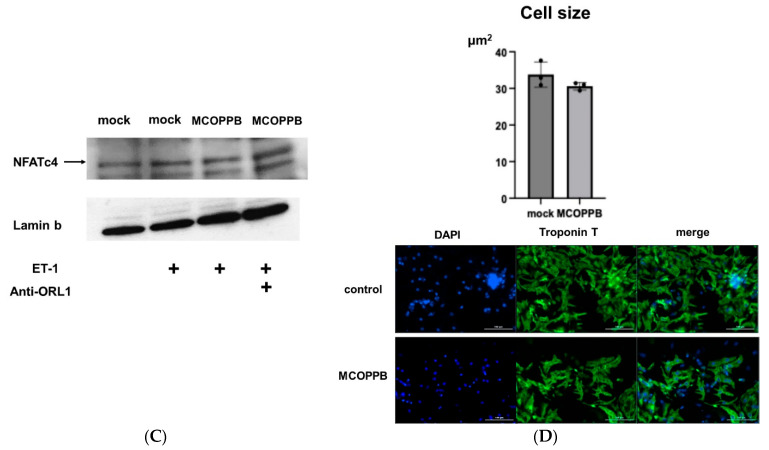
(**A**) ORL1 was identified in cardiac fibroblasts and cardiomyocytes. Primary adult rat brain tissue lysates, adult rat cardiac fibroblasts, neonatal rat cardiomyocytes (p2), and H9C2 cells (ATCC-CRL-1446) were immunostained with anti-nociceptin receptor antibody (alomone labs, AOR-015). Another set of the four cell groups were pre-incubated with ORL1-blocking peptide (alomone labs, BLP-OR015). (**B**) NPPA and NPPB were downregulated by MCOPPB. Neonatal rat cardiomyocytes were treated with a nociception agonist, MCOPPB (0.5 μM), or MCOPPB (0.5 μM) + anti-ORL1 (ORL1 antagonist, [Nphe1]Nociceptin(1-13)NH2]) (10 μM). All groups received ET-1 (100 nM). qPCR shows that NPPA and NPPB, which are downstream transcription genes of the NFAT signaling pathway and related to pathological hypertrophy, were downregulated by MCOPPB. Significantly, those downregulations by MCOPPB were diminished by adding the ORL1 antagonist. A two-tailed ANOVA with Bonferroni post hoc test was used. Primers for NPPA: forward, 5′-CGTATACAGTGCGGTGTCCAAC-3′; reverse, 5′-CATCTTCTCCTCCAGGTGGTCTAG-3′. Primers for NPPB: forward, 5′-AAGTCCTAGCCAGTCTCCAGAACA-3′. Reverse, 5′-TTGAGAGCTGTCTCTGAGCCATT-3′. (**C**) ORL1 inhibitor increased nuclear dephosphorylated NFAT. H9C2 cells were treated with MCOPPB (0.5 μM), ET-1 (100 nM), and anti-ORL1 (10 μM). Nuclear fractions of cells were extracted using an NE-PER nuclear reagents kit and analyzed by Western blotting with NFATc4 antibody (Santa Cruz Biotechnology—SC 271597). Lamin b (Santa Cruz Biotechnology—SC 374015) was used as a loading control. NFATc4 expression was significantly increased by adding the ORL1 antagonist. (**D**) Cardiomyocyte cell size was not changed with MCOPPB administration. Neonatal mouse cardiomyocytes (p3) were treated with MCOPPB (0.5 μM) at day 1 and day 2, and immunostained with troponin T at day 3. Cardiomyocyte imaging and an analysis of the images were conducted using the high-content imaging instrument Cytation 7 (BioTek). Average cardiomyocyte size was no treatment, 33.8 ± 3.4 μm^2^ vs. MCOPPB group, 30.6 ± 1.1 μm^2^, (*p* = 0.20, 3 plates/group). Representative images are shown (×200).

(1) Prior to in vitro experiments, we confirmed that the cardiac fibroblasts and cardiomyocytes contained ORL1 by Western blot (Figure 5A). Brain tissue lysates (positive control), rat cardiac fibroblasts, H9C2 cells, and neonatal rat cardiomyocytes all showed positive expression of the ORL1 antibody, and it was decreased by the addition of ORL1-blocking peptide.

(2) Neonatal rat cardiomyocytes were treated with the nociception agonist, MCOPPB (0.5 μM), ET-1 (100 nM), and anti-ORL1 (ORL1 antagonist, [Nphe1]Nociceptin(1-13)NH2]) (10 μM). qPCR showed that NPPA and NPPB were downregulated by MCOPPB. Significantly, those downregulations by MCOPPB were diminished by adding ORL1 antagonist (Figure 5B).

(3) H9C2 cells were treated with MCOPPB (0.5 μM), ET-1 (100 nM), and anti-ORL1 (10 μM). Nuclear fractions of cells were extracted using an NE-PER nuclear reagents kit and analyzed by Western blotting with NFATc4 antibody (Santa Cruz Biotechnology—SC 271597). NFATc4 expression increased by ET-1 was decreased by MCOPPB and significantly increased by adding the ORL1 antagonist (Figure 5C).

(4) Neonatal mouse cardiomyocytes were treated with MCOPPB (0.5 μM) at day 1 and day 2, and immunostained with troponin T at day 3. There was no difference between the no-treatment group and the MCOPPB-receiving group in cell size: no treatment vs. MCOPPB, 33.8 ± 3.4 vs. 30.6 ± 1.1 μm^2^, (*p* = 0.20, 3 plates/group) (Figure 5D). 

## 4. Discussion

Our in vivo experiments demonstrated that MCOPPB alone, without Gata4 overexpression, improved cardiac function, suppressed fibrosis, and induced angiogenesis, resulting in attenuating HF. In the echocardiographic analysis, there were three significant data points: (1) the ejection fraction improved; (2) the end-systolic volume decreased and stroke volume increased; and (3) the end-systolic wall thickness increased. Outcomes (1) and (2) suggest increased cardiac contractility, while (3) suggests cardiac hypertrophy. We measured heart weight at euthanasia, and found that heart weight adjusted by body weight increased in the MCOPPB group, specifically in the female group (Figure 3C). Additionally, immunohistochemistry analysis showed that cardiomyocyte diameter was increased. Since cardiac function was improved, we believe that the hypertrophy was not pathological, but rather physiological. The preliminary in vitro experiments showed that MCOPPB downregulated the pathological hypertrophy-related genes NPPA and NPPB [15] (Figure 5B). We also observed that nuclear NFAT was substantially increased by administering ORL1 inhibitor (Figure 5C). This suggests that the Calcineurin–NFAT pathway, which is an upstream signaling pathway of NPPA and NPPB [15], is downregulated by MCOPPB. Thus, our in vivo and in vitro results indicate that ORL1 receptor activation attenuated pathological cardiac hypertrophy.

Our next question is, “If MCOPPB downregulates the pathological hypertrophy pathway, does MCOPPB upregulate the physiological hypertrophy pathway?”. GPCRs are known to be a co-activator of receptor tyrosine kinase (RTK) [16]. The physiological hypertrophy inducer, the IGF receptor, belongs to RTK. Therefore, the ORL1 receptor, activated by MCOPPB, might co-activate the IGF receptor, which induces physiological hypertrophy. 

The VEGF receptor also belongs to RTK and the activated VEGF receptor induces angiogenesis. Our finding that MCOPPB induced angiogenesis also might be a result of the co-activation of the VEGF receptor by MCOPPB. Heineke J et al. [17] reported that Gata4 can enhance angiogenesis and suggested that the angiogenesis is in part through the regulation of the VEGF receptor. This report further supports our speculation that MCOPPB induces angiogenesis by cross-talk between the ORL1 receptor and VEGF receptor. Thus, we plan to investigate the dynamics of ORL1/IGF1 and ORL1/VEGF complex formation and effector protein recruitment.

Since the ORL1 receptor was identified in 1994, several researchers have investigated the effects of ORL1 activation on the cardiovascular system. It has been reported that intravenous and intracerebroventricular nociceptin administration produced hypotension and bradycardia [18,19]. It is not yet clear how the hemodynamic change is introduced by ORL1 activation, whether through the central nervous system or by direct ORL1 receptor activation on the cardiovascular cells. Since we did not measure blood pressure, it is also not clear whether hypotension contributed to the improvement of cardiac function. Our in vitro experiments suggest that the direct activation of the ORL1 receptor on the cardiomyocyte cell membrane induces molecular signaling pathway changes in the cell (Figure 5B,C). However, our in vitro cardiomyocyte size measurement showed that MCOPPB did not increase cell size (Figure 5D). Therefore, there is a possibility that cardiac hypertrophy is not induced by direct ORL1 activation on cardiomyocytes, but rather through changes in the central and/or peripheral nervous system. Since MCOPPB can pass through the blood–brain barrier [20], MCOPPB could activate the central nervous system. Thus, there are many questions to be answered about how MCOPPB improved cardiac function. At present, it is not clear how the ORL1 activator improves cardiac function in the ischemic heart and whether it is due to a systemic effect such as vasodilation and lower blood pressure, or by a direct effect on the heart. This mechanism needs to be clarified in order to establish ORL1 activation as a viable HF treatment.

Regarding gender differences, we analyzed whether the gender caused any effect on the echocardiographic longitudinal data, ejection fraction, wall thickness, and LV volume (Figure 2A–C). We did not find any significant differences. Since each gender group contained only three animals, we need to increase animal numbers to verify whether the difference in p values is significant.

Our goal is to translate the MCOPPB treatment into a clinically effective and safe therapy for combatting HF progression. Our in vivo experiment demonstrated that post-MI rats did not show any notable side effects such as body weight loss and lethargy with MCOPPB intraperitoneal injection for 3 months. Raffaele M et al. [13] administered MCOPPB (5 mg/kg/day) intraperitoneally to mice for 2 months to investigate if MCOPPB has anxiolytic and senolytic effects. They observed weight gain and mild hepatic stress characterized by a low grade of steatosis. In our study, we did not examine the liver. Regarding body weight, no significant weight gain in the MCOPPB group was observed compared to the saline group. In fact, weight gain in the male MCOPPB group was less than in the male saline group (% body weight gain; saline group vs. MCOPPB group: 22 ± 3% vs. 13 ± 0.6%, *p* < 0.05) (Figure 3B). We enrolled 8-week-old male and female rats. Although they are adults, they still continue to gain weight according to a weight chart (Charles River Laboratories website. https://www.criver.com/sites/default/files/resources/doc_a/CDIGSRatModelInformationSheet.pdf (accessed on 30 October 2024)).

Therefore, there is a possibility that our data indicate that MCOPPB suppressed weight gain in the male group. Another possible factor affecting body weight is the induction of myocardial infarction. In previous rat myocardial infarction experiments, we observed that some rats did not gain weight after the surgery, possibly because of the severity of the infarction. We plan to perform further experiments with prolonged administration of MCOPPB as well as increased animal numbers.

### Study Limitations

The preliminary in vitro experiments suggest underlying molecular mechanisms that enable MCOPPB to suppress HF. However, the experiments are incomplete. We used H9C2 cells and neonatal cardiomyocytes for the preliminary experiments. We plan to repeat thorough in vitro experiments with adult cardiomyocytes. We expect that further studies on the molecular mechanism of MCOPPB can also elucidate potential wanted and unwanted effects of its use.

## Figures and Tables

**Table 1:** Cardiac fibroblasts were treated with lentivirus encoding GFP or Gata4 for 14 days (*n* = 3). RNAseq data show that Gata4 upregulated the Pnoc gene 164-fold compared to GFP.

Gene Symbol	Name	Fold Change	*p* Value	FDR	Gata4_1	Gata4_2	Gata4_3	GFP_1	GFP_2	GFP_3
Pnoc	prepronociceptin	164.1469	0	0	103.0171	99.29082	91.93471	0.551751	0.677813	1.016347

## Data Availability

The original data will be made available upon request to the corresponding author, Megumi Mathison.

## Supplementary Materials

The following supporting information can be downloaded at: https://www.mdpi.com/2308-3425/11/11/355/s1, Table S1: raw echocardiographic data.
